# The Prophylaxis of Ophthalmia Neonatorum
*Abstract of a Paper read at a Meeting of the Bristol MedicoChirurgical Society on Wednesday, February 9th, 1927.


**Published:** 1927

**Authors:** A. E. Iles

**Affiliations:** Surgeon, Bristol Eye Hospital; Ophthalmic Surgeon, Bristol General Hospital


### THE PROPHYLAXIS OF OPHTHALMIA NEONATORUM.*

BY

A. E. Iles, O.B.E., M.B., F.R.C.S. Eng.,

*Surgeon, Bristol Eye Hospital;*
*Ophthalmic Surgeon, Bristol General Hospital.*

---

Ophthalmia neonatorum, before compulsory notification became law, accounted for over one-third of all cases at the local Blind Institutions. Since the enforcement of notification there has been a notable diminution in cases of blindness, though the fall in the total number of cases of the disease has been less. The following figures show the local incidence of the disease.

| Year. | Number of cases notified. | | Cases of damage to sight. | |
|---|---|---|---|---|
| | Total. | Per 1,000 births. | Total. | Partial. |
| 1922 | 119 | 15·7 | — | 4 |
| 1923 | 82 | 11·3 | — | 3 |
| 1924 | 89 | 12·5 | — | 1 |
| 1925 | 58 | 8·3 | 1 eye | — |

The diminution may be ascribed to three causes :—

1. Training and registration of midwives.
2. Increased use of Crédé's method of prophylaxis.
3. Compulsory notification, leading to early treatment.

* Abstract of a Paper read at a Meeting of the Bristol Medico-Chirurgical Society on Wednesday, February 9th, 1927.

In man the lids are separate before birth. Though the eyes are not opened in the vagina, the lashes and lids may become contaminated. It is therefore essential that the lids should be thoroughly cleansed before the eyes are opened. Care must be taken that the bath water does not get into the eyes. The conjunctival sac makes an ideal incubation chamber. By the use of prophylactic methods the incidence of the disease has been reduced practically to zero in English lying-in hospitals (on the continent Kostling states 9 per cent. to 0·65 per cent.).

Disease arising from birth infection is manifest during the first five days, 50 per cent. of all cases occurring on the third day. Disease starting later than this is due to subsequent infection. Various organisms are concerned, but the severe form is due to the gonococcus. The organisms rapidly penetrate into the epithelium.

Among drugs that have been used in prophylaxis one may mention :—

Silver Nitrate 2 per cent., Crédé's original method, or 1 per cent. as used at the General Hospital.

Silver Acetate 1 per cent., because this is a saturated solution at room temperature, so that accidental concentration is avoided.

Protargol or Argyrol 5 per cent.

Mercuric Chloride 1/2000.

Mercurochrome 1 per cent.

Silver salts are said to stimulate the early development of lymph tissue in the fornices where it is not present at birth. The escharotic action of the salts is probably of value in the very earliest phase of infection, before the organisms have penetrated deeply. Protargol is slightly more efficacious than argyrol ; in treatment of purulent conjunctivitis 33 per cent. protargol has been found more germicidal than 2 per cent. silver nitrate. For prophylactic use the nitrate unfortunately produces a definite reaction, so that its routine use is shirked. As regards reaction only, mercurochrome has a material advantage, as the following figures show:—

| 1 per cent. solution of | Number of cases showing a reaction. | | No Reaction. | Total. |
|---|---|---|---|---|
| | Severe. | Slight. | | |
| Silver Nitrate.. | 30 | 93 | 30 | 153 |
| Mercurochrome | 5 | 17 | 131 | 153 |

As regards treatment, once the disease has occurred I have hitherto employed protargol 25 per cent. Silver nitrate must be used with great care, and only for painting the lids. All these drugs entail frequent irrigation during treatment. This is avoided with mercurochrome 15 per cent., which penetrates deeply and will act on the gonococci in the tissues.

During the past seven months we have had ten severe and thirteen slight cases of ophthalmia neonatorum at the Eye Hospital. For all these the treatment has been irrigation with 1/10,000 perchloride of mercury and painting with 15 per cent. mercurochrome; drops of 1 per cent. of the latter were given for use at home. In only one case did we have to paint a second time; in no case was the second eye, protected by the 1 per cent. drops, infected from the first. We were able to dispense with frequent irrigation, and in only one case did we have to admit a child: this baby's eyes had been burnt by frequent applications of silver nitrate 1 per cent. before it was brought up. Discharge and swelling rapidly disappear under the influence of this treatment, and the cornea is not damaged. In late cases, however, we found that 2 per cent. silver nitrate was superior. The number of cases in which gonococci were found is yet too small for a comparison of efficacy in established gonococcal ophthalmia.

### SUMMARY.

Mercurochrome is as efficient as silver nitrate in the prophylaxis of ophthalmia neonatorum, it is safer, and is devoid of reaction.

In the treatment of established conjunctivitis with mercurochrome we see rapid subsidence of swelling and discharge. The necessity for frequent irrigation, and so for admission of the baby to hospital, is avoided. The duration of treatment is reduced, and the unaffected eye is protected.

